# Near Infrared Efficiency Enhancement of Silicon Photodiodes by Integration of Metal Nanostructures Supporting Surface Plasmon Polaritrons

**DOI:** 10.3390/s23020856

**Published:** 2023-01-11

**Authors:** Elia Scattolo, Alessandro Cian, Luisa Petti, Paolo Lugli, Damiano Giubertoni, Giovanni Paternoster

**Affiliations:** 1 Sensors and Devices Center, Bruno Kessler Foundation, I-38123 Trento, Italy; 2 Faculty of Science and Technology, Free University of Bozen, 39100 Bolzano, Italy

**Keywords:** plasmonics, plasmonics photodetector, focused ion beam, silicon photodiode, near-infrared, LiDAR

## Abstract

Recent years have witnessed a growing interest in detectors capable of detecting single photons in the near-infrared (NIR), mainly due to the emergence of new applications such as light detection and ranging (LiDAR) for, e.g., autonomous driving. A silicon single-photon avalanche diode is surely one of the most interesting and available technologies, although it yields a low efficiency due to the low absorption coefficient of Si in the NIR. Here, we aim at overcoming this limitation through the integration of complementary metal–oxide–semiconductor (CMOS) -compatible nanostructures on silicon photodetectors. Specifically, we utilize silver grating arrays supporting surface plasmons polaritons (SPPs) to superficially confine the incoming NIR photons and therefore to increase the probability of photons generating an electron-hole pair. First, the plasmonic silver array is geometrically designed using time domain simulation software to achieve maximum detector performance at 950 nm. Then, a plasmonic silver array characterized by a pitch of 535 nm, a dot width of 428 nm, and a metal thickness of 110 nm is integrated by means of the focused ion beam technique on the detector. Finally, the integrated detector is electro-optically characterized, demonstrating a QE of 13% at 950 nm, 2.2 times higher than the reference. This result suggests the realization of a silicon device capable of detecting single NIR photons, at a low cost and with compatibility with standard CMOS technology platforms.

## 1. Introduction

Silicon-based photodetectors are a well-established and commonly utilized technology due to their convenient manufacturing cost, their high efficiency, and their process compatibility with the complementary metal–oxide–semiconductor (CMOS) process. Recently, they have also been employed for near-infrared (NIR) light detection, due to the growing interest towards emerging applications such as quantum distribution key [1], gas sensing [2,3], time-correlated single photon counting [4,5] and light detection and ranging (LiDAR) for autonomous driving [6]. Usually, in the majority of these applications, a NIR laser is used as a light source to illuminate the surrounding environment, with a characteristic wavelength in the range (900–1000) nm, which is chosen based on some constraints like human safety and the sun’s background light filtration.

Additionally, most of these applications call for detectors able to provide single-photon resolution, leading to the need to utilize single-photon avalanche diodes (SPADs) and photomultipliers (PMs) platforms. In particular, SPADs are devices designed to operate above their avalanche breakdown voltage in the Geiger mode, where a single photon can be converted into a quantifiable electrical signal thanks to the avalanche generated via a phenomenon called impact ionization. While silicon-based SPADs rely on well-developed and known silicon technologies that lead to a lower fabrication cost, SPASs based on III-V materials present a high photon detection, but higher design and fabrication costs.

For design reasons, SPADs and SiPMs are commonly fabricated on epitaxial substrates, with active thicknesses typically in the range of a few micrometers [7]. Because of the low absorption coefficient of Si in the NIR, Si SPADs and SiPMs s have a photon detection efficiency (PDE, the probability that an incoming photon on the SiPM surface is detected producing an output pulse) of only a few percentage points in the NIR. The scientific community has tried to overcome this drawback by using thicker epitaxial silicon substrates (up to 10 μm). This resulted in PDE of 10% at 950 nm [8], although this poses problems with the design of SiPMs and SPADs with small cells (less than 25 μm), and performance in terms of single photon temporal resolution [9].

Recently, other approaches have been proposed by the scientific community: (i) light trapping (by means of nanotexturing) [10]; (ii) integration with photonic structures (metalenses or metasurfaces) [11], or (iii) integration with metal nanostructures supporting plasmonic resonances, [12,13]. Among the others, a very interesting proposed solution is the integration of thin photodetectors with a nanostructure supporting surface plasmon polaritons (SPP) able to confine the light in a sub-wavelength region at the detector surface, with a subsequent absorption enhancement.

In the last twenty years, many theoretical and experimental studies have explored the attractive optical properties of photonic and plasmonic nanostructures [14,15,16,17], especially in terms of light confinement for advanced sensing. In fact, the SPP light confinement has further advantages in terms of fast timing and reduced dark noise, thanks to the use of ultra-thin silicon detectors.

A comprehensive comparison of the results obtained in this field is complicated by the fact that the performance depends on many factors like the depth of the device junction, the semiconductor material (silicon, group III-V materials, silicon-on-insulator) and the presence of back reflection layers (with the latter providing better performance).

These variables render the result obtained in this work difficult to systematically compare with others previously reported. Anyway, we tried to discuss more in-depth the results previously obtained by the scientific community and to compare them to our devices, when possible, underlying the differences in terms of structure, materials or target wavelength. Ref. [18] reported an enhancement of the light sensitivity in silicon-on-insulator (SOI) p-n junction devices coupled with metal gratings, made of Au, Al and Ag. In this study, the enhancement is mainly due to the coupling of the diffracted light by the grating with the modes in the SOI waveguide. They reported a theoretical study for a limited range of grating pitch values 260– 340 nm, obtaining a quantum efficiency (QE, i.e., the probability of generation and collection of an electro–hole pair per and absorbed incoming photon) peak centered around 700 nm of wavelength. For the Ag/Ti grating with a periodicity of 300 nm and a thickness of (103/5) nm, they obtained a value of external quantum efficiency (EQE, i.e., the probability of generation of an electron–hole pair per and absorbed incoming photon) of 22% at 700 nm, many times greater than the reference value of a few percentage points (less than 2%). In [19], the authors achieved a narrowband photodetection in the near-infrared (at 1450 nm) by means of a Schottky photodetector integrating 1D Au grating, thanks to a plasmon-induced hot electron generation in the metal grating. The Au thickness is of 200 nm, with a constant slit width of 250 nm and increasing pitch values, from 800 nm to 1100 nm. Because of the higher periodicity, peaks are centered at longer wavelengths (greater than 950 nm), starting from 1300 nm and moving toward 1650 nm. In [19], the authors reported a spectral responsivity of about 0.0005 $\frac{A}{W}$. Such a value, even if considered remarkable, because it is obtained at sub-bandgap energy, is still too low to be exploited in real applications. Finally, in [20], the authors proposed Au/Ti 1D grating with a thickness of 35 nm, a periodicity of 200 nm and a duty cycle (DC, i.e., ratio between the width and the periodicity of the grating) of 0.50, to enhance a resonant-cavity subwavelength metal–semiconductor–metal photodetector. To promote the enhancement, a quarter wave stack multilayer Bragg reflector is placed at the bottom of the device. By using a GaAs semiconductor absorber, they reached an EQE of about 28% at 810 nm, five times more compared to the reference device EQE (5%).

It is difficult to find a study in which the authors demonstrate NIR light confinement by CMOS-compatible nanostructures on a crystalline silicon substrate (not SOI), as the first approach for future integration on single-photon detectors like SPADs and SiPMs. Thence, the idea of this work: the integration on silicon photodiode (Si-PD) of CMOS-compatible nanostructures, specifically, metallic grating supporting SPPs, to confine superficially the incoming NIR photons and therefore increase the probability of photons generating an electron–hole pair.

The fabrication of the proposed idea needs to deal with the integration of the metallic array nanostructures on top of the device’s active region. Typically, it is also required that the metal structures lie very close to the active substrate (a few nanometers) to optimize the coupling between the resonant structures and the device. Contrary to the fabrication on inert substrates, this task faces several challenges: surface topography, materials inter-compatibility, thermal budget limitations, as well as side damages induced by the integration, which could degrade the performance of the sensor, thus the choice of a CMOS-compatible nanofabrication technique becomes crucial. The focused ion beam (FIB) nanofabrication technique is one of the most promising in terms of feasible geometries and rapid prototyping. However, the direct patterning by FIB could induce ion implantation in the substrate or lattice damage and sputter. Both of them can damage the sensor or affect its electrical performances [21,22,23,24].

In this work, we demonstrate the integration of metallic nanogratings supporting SPP, on the top of thin Si-PDs produced with a CMOS-like process flow. The Si-PDs used in this work have been fabricated at Fondazione Bruno Kessler, by using a technological platform similar to the one used for the production of SiPMs (FBK NUV-HD SiPMs [25]). Si-PDs have been fabricated on 3 μm-thick epitaxial silicon, and they feature a junction depth of about 300 nm. For Si-PDs with this specification, the QE at 950 nm is around 5%.

In order to realize the proposed idea and thus ensure higher absorption of Si-PD in the NIR, specifically at 950 nm, the geometry of the plasmonic metal array was tuned by exploiting finite difference time domain (FDTD) simulations (Lumerical [26]) to achieve maximum diode performance at 950 nm through light confinement. Thereafter, the plasmonic metal array is integrated on the active area of a Si-PD by a finely-tuned FIB patterning process obtaining a plasmonic silver array with a pitch of 537 nm, a DC of 0.84 and a metal thickness of 110 nm. Finally, the integrated PD is characterized in terms of electro-optical response to assess the performance, demonstrating a QE of 12% at 950 nm.

## 2. Detector Design and Optical Simulations

**Figure 1:**
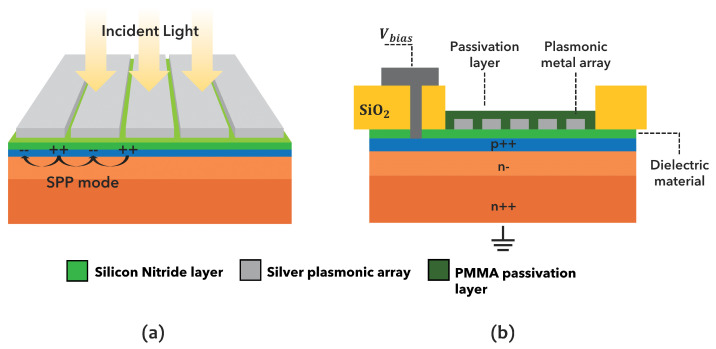
(**a**) Three-dimensional sketch of the proposed structure of plasmonic metal array fabricated on the top of a silicon photodiode passivated with a thin dielectric film and supporting surface plasmon polaritons (SPP) mode, and (**b**) cross-section of the plasmonic metal array over the active area of the Si-PD (p++/n-/n++), with the Si-PD passivated by a thick SiO_2_ layer. The bias voltage ($V_{bias}$) is applied from the metal top contact, while the Si-PD is grounded from the bottom.

The proposed sensor design is shown in Figure 1, in which both the 3D sketch and the cross-section are reported. The device consists of a silicon epitaxial substrate (n- on n+) with a shallow planar p-n junction (in blue), i.e., the interface between p-doped (excess of holes) and n-doped (excess of electrons) silicon, thus allowing the electrical current to flow only in one direction. The surface is protected by a thin layer of dielectric material (in green). A 1-dimensional metal array (in grey) is then placed just above the dielectric layer, close to the active p-n junction. Such a structure is well known in the literature to support SPP modes at the metal/dielectric interface, along the sensor surface, in a direction perpendicular to the slits (as represented by the black arrows) [27]. These SPPs also have the characteristics to be strongly confined along the direction perpendicular to the sensor surface.

The periphery of the device is passivated by thick SiO_2_ film, while the bias voltage ($V_{bias}$) is applied from the metal top contact and the Si-PD is grounded from the bottom.

The resonance frequency of the metal grating depends on several geometrical parameters of the structure (grating pitch, metal and dielectric material, metal thickness). In order to tune the grating geometry for our specific purpose (efficiency enhancement at 950 nm), finite element optical simulations have been run by means of the Lumerical FDTD.

### 2.1. Simulation Methods

In the simulation environment, the Si-PD is represented by a semi-infinite silicon slab, over which a thin dielectric film is placed. The dielectric layer has a twofold goal: (i) passivating the detector surface, reducing the surface recombination velocity, and (ii) enabling SPP excitation at the interface between metal and a dielectric material. It is worth noting that to maximize the coupling between the surface SPP and the silicon, enhancing the carrier generation in the active substrate, the dielectric layer thickness has to be extremely thin (order of 10 nm) and precisely tuned, as demonstrated by numerical simulations in the next section.

**Figure 2:**
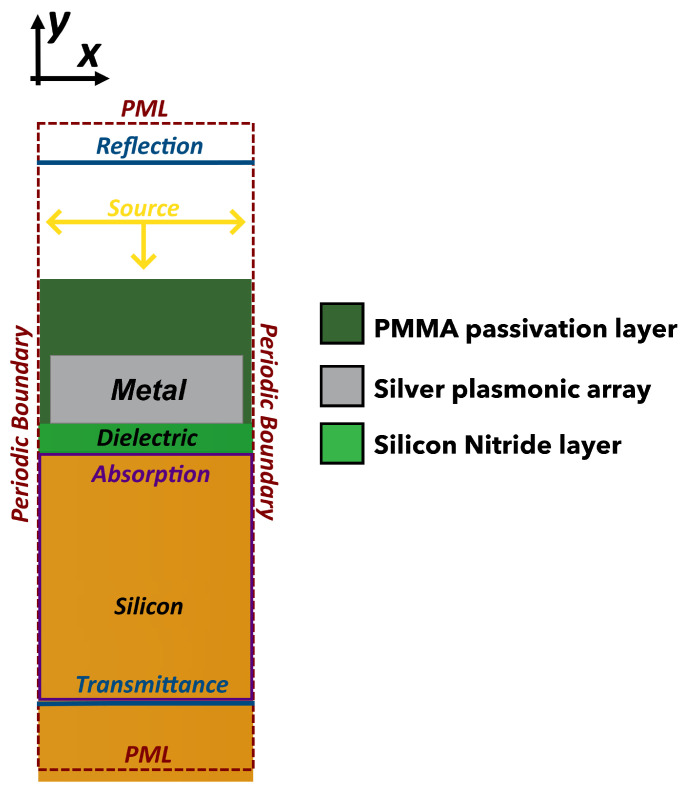
Cross section of the simulated structure cell with boundary conditions (red dotted line) and recording monitors (blue for reflection and transmittance, purple for absorption).

The simulated structures consist of 1-dimensional gratings, modeled as an array of infinite-length nanostrips, see Figure 1a. The light source is a planar wave TM polarized with respect to the strips. Only a unit cell has been studied by setting the proper boundary conditions to replicate an infinite array, as shown in Figure 2. To maximize the fraction of the absorbed power in a 3 μm thick Si at 950 nm, the following parameters have been finely tuned (see in the next section): (i) grating pitch; (ii) dielectric thickness; (iii) metal thickness; (iv) duty cycle (defined as the ratio between dot width and pitch). The parameter that was maximized is the total absorption power fraction at 950 nm, calculated as the ratio between the power absorbed in the first 3 μm of silicon (the active thickness of our devices) versus the total incident power.

**Figure 3:**
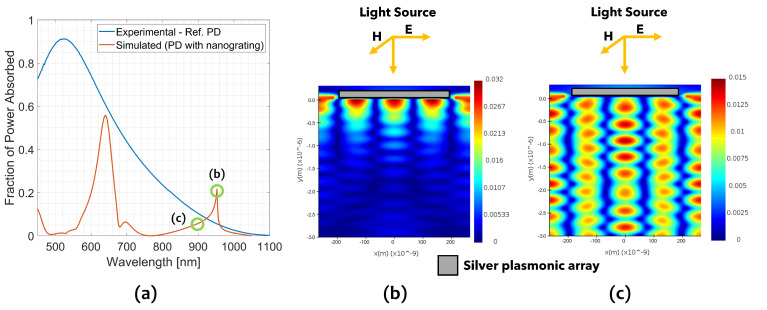
(**a**) Simulated absorbed light (orange solid line) and reference spectra (blue solid line) as a function of wavelength in the range (450–1100) nm; (**b**) magnetic field intensity profile of the simulated section in Figure 2 at 950 nm; and (**c**) magnetic field intensity profile of the simulated section in Figure 2 at 880 nm demonstrating a diffraction pattern.

Figure 3 reports the simulated total absorbed power fraction versus the wavelength in the full visible-NIR spectrum (orange curve). The same curve calculated for a device without any metal grating on the top is also reported for comparison (blue solid line). The simulated spectrum features three main peaks: (i) an intense peak of around 58% at 640 nm, (ii) a less intense peak of 8% at 700 nm and (iii) the peak of our interest of 22% at 950 nm.

The geometrical parameters used in the simulated structure of Figure 3a have been calculated and tuned by means of a simulative campaign, as described in Section 2.2, in order to maximize the absorption at 950 nm in the first 3 μm of silicon. The silver grating has a thickness of 110 nm, a pitch of 535 nm and a duty cycle (DC) of 0.80, while the dielectric interposer is a 11 nm thick silicon nitride layer. Finally, a 220 nm thick layer of Polymethyl methacrylate (PMMA) coats the silver grating. The latter was designed to protect the metal and prevent silver tarnishing.

It is worth noting that the simulated absorption curve shows a lower absorption than the reference across the whole simulated spectrum (450–1100) nm, with the exception of the peak at 950 nm where the absorption reaches the 22%, about 4.4 times higher with respect to the reference.

Figure 3b,c reports the H field 2D-maps, calculated on a plane perpendicular to the sensor surface, at 950 nm (green circle marker labeled with (b)) and at 900 nm (green circle marker labeled with (c)), respectively. The magnetic field at 950 nm shows a pattern compatible with a SPP quadrupole resonance, with an evanescent component of the electromagnetic field, which becomes strongly confined in the first 500 nm from the surface. The EM field confinement can be considered mainly responsible for the enhanced absorption at 950 nm. At a wavelength far from this resonance (i.e., at 900 nm), the electromagnetic field penetrates much deeper into the substrate, passing through the full active thickness of the device.

Generally speaking, the resonant modes of an open metal grating consist of hybrid optical-plasmonic resonance modes due to a combination of different phenomena. Among the others: (i) cavity mode resonance in the inter-strip gaps [20,28]; (ii) already mentioned SPP resonance at the interface metal/dielectric and (iii) Wood–Rayleigh (WR) anomaly that consists in an abrupt change in transmittance at a wavelength, where an order of diffraction is reflected outside the detector plane [29]. The latter effect is considered responsible for the asymmetric shape of the absorption peak at 950 nm. Approaching the WR anomaly at 950 nm, the first light diffraction order through the grating becomes more and more randent to the surface, producing a strong coupling with SPP. Then, at the WR anomaly wavelength, the diffraction order is reflected out of the silicon surface, producing an abrupt drop in the absorption. For the sake of completion, the physical explanation of the peak at 950 nm is well described and detailed in [30].

### 2.2. Simulative Campaign

The tuning process was a challenging task because of the multi-variable structure, in which each factor is not independent of the other. The first step is to choose the best metallic and dielectric materials in terms of optical properties and their CMOS compatibility. The metal and dielectric materials available in Fondazione Bruno Kessler facilities and compatible with the PD fabrication process are silver, gold, aluminum, silicon nitride and silicon oxide, respectively. Among metals, silver is the most promising option because (i) it has the strongest plasmonic resonance at 950 nm, reaching an absorption 4.4 times more than the reference (compared to Au and Al, which show an enhancement of 2.1 and 1.9, respectively), (ii) contrary to Au, Ag metal does not require any adhesion layer that could modify the plasmonic resonance behavior. On the other hand, silver is prone to tarnishing, and must be protected from the external environment. Regarding the dielectric material, both SiO_2_ and Si_3_N_4_ have been tested as interposer materials between silicon and metal grid in the simulations. In both cases, thicknesses in the range of 5–50 nm have been tested. Using SiO_2_ leads to an optimal thickness of 4 nm only, too thin to be precisely fabricated and to be used as a passivation layer for the PD. In the case of Si_3_N_4_, the maximum absorption at 950 nm is reached with a thickness of 11 nm. Therefore, Si_3_N_4_ can be considered the better choice, mainly taking into account manufacturing feasibility.

**Figure 4:**
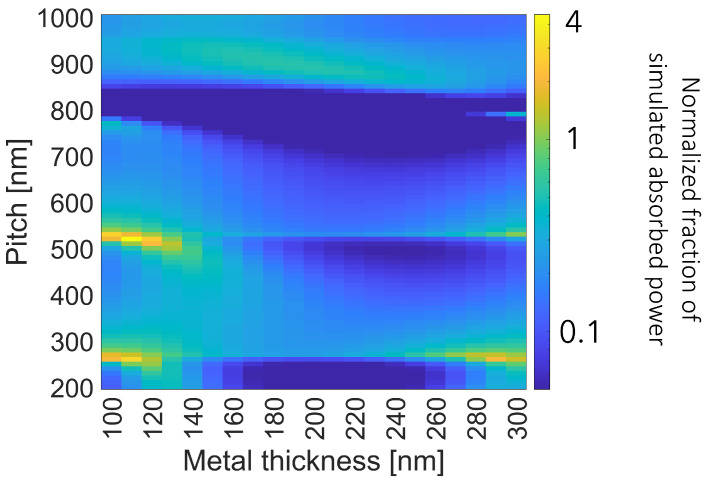
Normalized fraction of simulated absorbed power over the ideal absorption (with null reflection) of a 3 μm silicon slab integrated with a plasmonic metal array of a DC of 0.80 over a thin Si_3_N_4_ layer of 11 nm at the wavelength of 950 nm as a function of the pitch and metal thickness.

**Figure 5:**
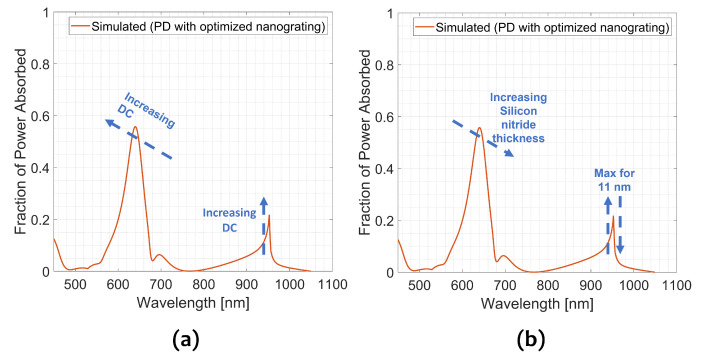
Fraction of simulated absorbed power as a function of wavelength and of the duty cycle (DC) in (**a**) and of the silicon nitride thickness in (**b**).

Once the materials have been chosen, the geometric parameters (grating pitch, Si_3_N_4_ thickness, Ag thickness and duty cycle) need to be tuned. In spite of the multivariate problem, the tuning process has been faced by changing only one parameter at a time in an iterative process. The following steps could be summarized:

1. Find the pair of Ag thickness and grating periodicity (pitch) values that provides the highest absorption at 950 nm, see Figure 4;
2. Select value pairs that are feasible by the nanofabrication point of view (low aspect to ratio factor) and at the same time guarantee a high absorption at 950 nm;
3. Study the influence of Si_3_N_4_ thickness and DC on the fraction of light absorbed at 950 nm and select the best values, see Figure 5.

#### 2.2.1. Ag Thickness and Grating Pitch

The tuning process aimed at maximizing the absorbed power fraction of the structures in the first 3 μm of silicon at 950 nm. In the following plots, this value has been normalized over the ideal absorption (with null reflection) of a 3 μm silicon without any metal grating on the top (absorption of about 5% at 950 nm). Figure 4 reports the normalized absorbed power fraction as a function of the grating thickness and pitch, from 100 nm to 300 nm the first and from 200 nm to 1000 nm the latter. At the pitch values of 260 nm, 535 nm and 790 nm, a higher absorption is visible. This effect is probably related to the WR anomaly at the n-th order of diffraction. In particular, at these pitch values, a strong maximum is present when the Ag thickness reaches values around 110 nm and 290 nm.

Considering the nature of the focused ion beam (FIB) technique (accelerated ion beam focused on sample to mill the material), a higher Ag thickness means higher dwell time (duration of stay unblanked over a point [μm]), leading to a larger milling spot. As a result, the thicker the Ag layer is, the more difficult it is to obtain narrow slits. Thence, among the cited values couples the preferred Ag thickness is 110 nm with a pitch of 535 nm.

#### 2.2.2. Si_3_N_4_ Thickness and Duty Cycle

The influence of the Si_3_N_4_ thickness and DC is studied in Figure 5, where the absorbed power fraction as a function of the wavelength is reported.

Concerning the influence of DC (Figure 5a), the peak position at 650 nm has a blueshift for increased DC and a slightly increased intensity. On the contrary, the behavior of the peak at 950 nm is not influenced by the DC, but the higher the DC is, the more intense the peak is. Thence, theoretically, the best DC is 0.95, corresponding to a narrow slit of about 30 nm. To enable a feasible and reproducible nanofabrication process, a DC of 0.80 (slit of about 100 nm) has been chosen as the best compromise between performance and feasibility.

Regarding the thickness of Si_3_N_4_ (Figure 5b), the position of the peak at 650 nm has a redshift and a decrease in intensity by increasing thickness. The position of the peak at 950 nm is not affected by thickness, but its intensity does not have a monotonic dependence on the thickness of Si_3_N_4_, having indeed its maximum at 11 nm. The intensity of the peak at 950 nm is very sensitive to the thickness of Si_3_N_4_, in fact, the efficiency drops down to 4% only for thickness greater than 18 nm and lower than 5 nm.

To summarize, the optimized structure features a silver grating with a thickness of 110 nm, a periodicity of 535 nm and a DC of 0.80 lying over a thin 11 nm silicon nitride layer, with the array being passivated by 220 nm of PMMA.

## 3. Micro- and Nano-Fabrication of Integrated Plasmonic Detectors

Si-PDs were fabricated using a standard CMOS-like process at the internal facility of Fondazione Bruno Kessler. They were produced using a similar manufacturing process as the one developed for near-ultraviolet—high density (NUV-HD) SiPM technology, well described in [31,32]. Such a structure is provided with a shallow p on n junction, optimized to promote the photon detection efficiency in the near ultraviolet (NUV) spectral range. In fact, one of the challenges of this work is to demonstrate the potential efficiency enhancement of an optimized NUV device in the NIR by means of light confinement through plasmonic nanostructures.

### 3.1. Micro-Nano Fabrication Methods

In this design, the Si-PD consists of a thin n-type epi-silicon layer (nominal thickness of 3 μm thick, featuring a planar shallow p-n junction (in order of (200–300) nm deep), formed by means of 11B ion-implantation. Subsequently, the top silicon surface is passivated by depositing the thin Si_3_N_4_ layer (by means of CVD deposition) and the periphery of the PD is protected by a thick (about 500 nm) layer of silicon oxide. The ohmic contact is opened through this layer, and an aluminum metallization is deposited to form front contacts to the p-type silicon to bias the junction.

To fabricate the silver nanostructures on top of the Si-PD, the Ag layer has been evaporated in an ultra-high vacuum physical vapour deposition (PVD) over the active area of the Si-PDs (100 × 100 μm${}^{2}$). Areas were defined by electron beam lithography (EBL), using a 10 keV electron beam on positive tone resist (PMMA). After the development, the Ag areas of Si-PDs were finally defined by removing the unexposed resist by lift-off in warm acetone (35 ${}^{\circ }C$). Successively, the plasmonic nanostructures were patterned by FIB. After the patterning, to avoid silver oxidation and tarnishing, as well as the consequent degradation of the plasmonic performances, the device surface is capped with 220 nm of PMMA film by spin coating. The PMMA capping is then removed from the photodiode’s pads with another EBL and development step, to make the pads electrically connectable to the printed circuit board (PCB) by gold-wire bonding.

FIB patterning consists of an accelerated ion beam focused on the sample substrate. Because of the energy gain, the ions are able to sputter the sample’s surface atoms, hence milling the surface. The milling efficiency, called sputter yield (Y, i.e., the number of sputtered atoms per incident ions), is affected by the sample’s material as well as by the beam features (energy and ion species). Because the FIB process does not require any tool to transfer the pattern (unlike EBL and photolithography, which need a resist and the latter also a hard mask), the geometries achievable by FIB have almost no constraints. However, the direct patterning by FIB could induce ion implantations in the substrate creating defects and damages in the sensor [33].

The Ag plasmonic nanostructures were patterned by using a Raith Velion FIB-SEM equipped with an Au-Si liquid metal alloy ion source (LMAIS) [34]. The structures were defined by an Au ion beam, accelerated at 35 keV with an ion current of 23 pA, and with the ion beam normal incidence to allow a better lateral resolution.

### 3.2. Experimental Activities

Contrary to the fabrication on inert substrates, the integration of plasmonic nanostructures on an active device by FIB needs to consider the surface topography, the device’s composition, the thermal budget limitations, as well as the ion implantation. Thence, the FIB milling parameters have been tuned by: (i) optical investigation of the structures by secondary electron microscope (SEM) images, (ii) selection of the optimal ion fluence (number of ions irradiated per unit of surface, also expressed as total charge irradiated per surface unit [μC/$cm^{2}$]) value to remove Ag completely by atomic force microscope (AFM) and (iii) evaluation of the volume fraction of the implanted gold in the underlying device by dynamic secondary ion mass spectrometry (SIMS). The presentation of the FIB optimization process is not one of the aims of this work, for further details see [35].

The FIB process tuning leads to the following patterning parameters set: beam energy of 35 keV, beam current of 23 pA, and ion fluence of 10,000 $\mu C/cm^{2}$.

**Figure 6:**
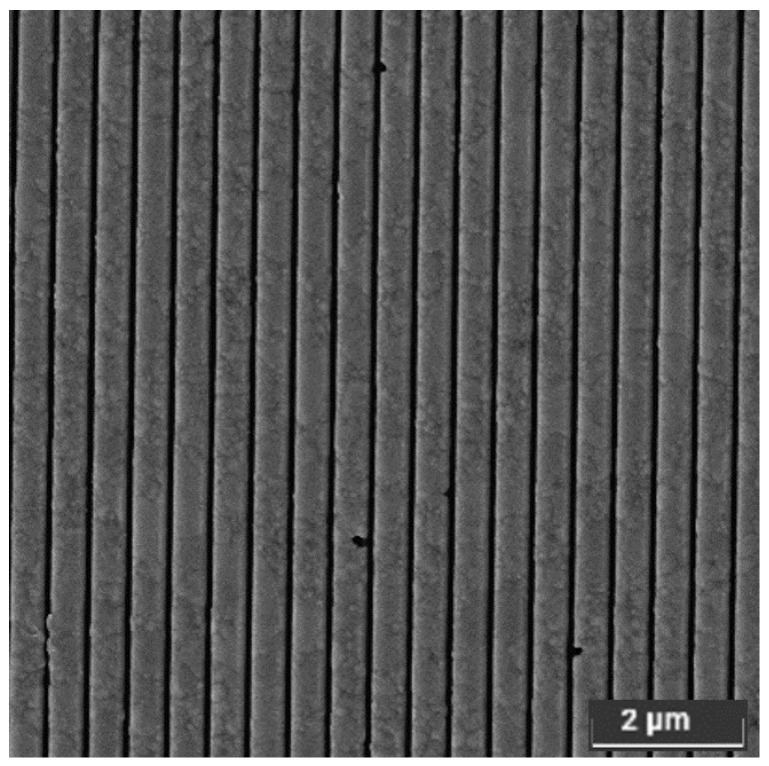
Top view SEM image of the FIB patterned 1D silver grating characterized by a pitch of (537±5) nm, a dot width of (451±4) nm and therefore a DC of 0.84.

**Figure 7:**
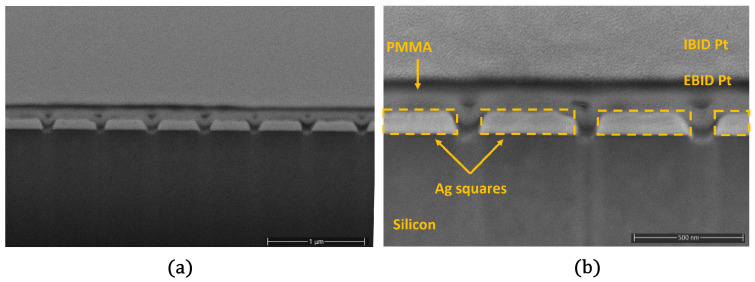
(**a**) Cross-section SEM image of the FIB patterned 1D silver grating covered by 50 nm of electron beam induced Pt deposition (EBID), and 1 μm of ion beam induced Pt deposition (IBID). (**b**) Zoom-in of the cross-section SEM image of the FIB patterned 1D silver grating with labels indicating precisely the different objects in the cross-section.

Figure 6 reports the top view SEM image of the 1D Ag grating integrated on a Si-PD by FIB optimized process. The plasmonic array consists of nanostrips with a measured pitch of (537±5) nm compatible with the nominal value, a dot width of (451±4) nm and therefore an average DC of 0.84. In Figure 6, Ag grains are clearly visible and in some places have been blown away, probably due to too much stress. In addition to the top view image, the cross-section SEM image of a 1D grating is reported in Figure 7.

The 1D grating reported in Figure 7a was produced with the same FIB process parameters but on a blank substrate (silicon chip) to enable faster analysis. The cross-section of the fabricated nanoarray was acquired using Helios PFIB-SEM—equipment from Thermofisher. To avoid any damage to the Ag structures, a platinum layer was deposited using a gas injection system, 50 nm electron beam induced deposition (EBID), and 1 μm ion beam induced deposition (IBID), before proceeding with the cross section by a 30 keV Xe+ beam.

The cross-section image confirms the thickness of the Ag layer of 110 nm and suggests the funnel profile shape of the grating slit as a consequence of the nature of the FIB process. In addition, at the bottom of the slits, a brighter region is present as confirmation of the fraction of Au atoms implanted in the underlying device. For the sake of completeness, Figure 7b shows all the labels to precisely indicate the different layers in the cross-section.

## 4. Electro-Optical Device Characterization

### 4.1. Electro-Optical Characterization Methods

**Figure 8:**
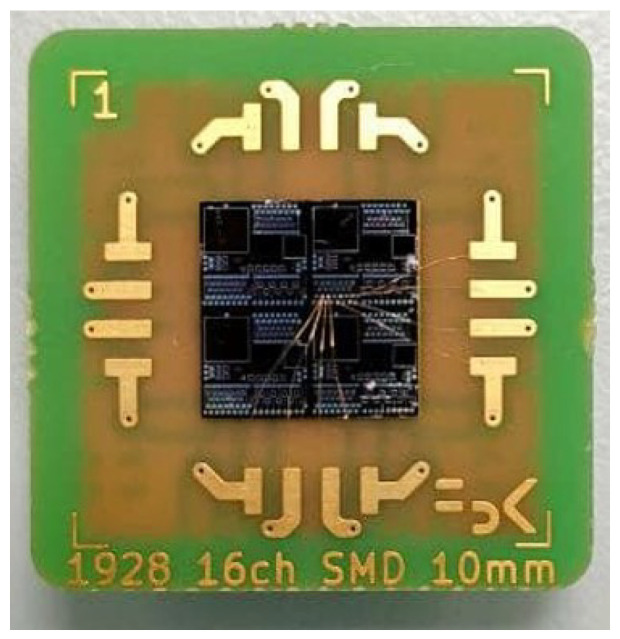
Top view picture of a produced sample after the integration on a printed circuit board (PCB).

To characterize the integrated Si-PD and to perform the electro-optical characterization, the Si-PD has been packaged on an appropriate printed circuit board (PCB), see Figure 8. The electrical link between the Si-PD’s metal pads and the PCB pins is made via the Au wire bonding technique. The back of the chip is coated with metal to act as bulk contact, yet, the chip is glued to the PCB by using conductive epoxy glow, with a final thermal curing process at 80 ${}^{\circ }$C.

Si-PD has been characterized using a custom-developed optical setup, which uses a halogen lamp as the (unpolarized) light source and a grating monochromator to choose the light wavelength impinging on the samples. The light exiting the monochromator passes through a variable aperture slit, which is set to select a bandwidth of 3 nm around the desired wavelength. This defines the wavelength resolution of the characterization setup. A polarizing filter is then mounted after the slit, to select a TM polarization with respect to the grating, for a direct comparison of the experimental spectrum with the simulations. The samples are mounted on a 3D moving holder that grants a precise positioning of the devices with respect to the incident light beam. Once the PCB pins are connected to the holder, the spectral responsivity (SR, i.e., the ratio of generated photocurrent and incident optical power determined in the linear region of response), as well as the QE can be calculated. The Si-PD is biased at 0 V working in photovoltaic mode.

Responsivity spectra are acquired as a function of the incident wavelength, within the (450–1100) nm range. The QE of a reference diode, produced on the same chip, with the same thin layer of Si_3_N_4_, but without any metal grid has also been measured for direct comparison. These spectra can be compared to the expected simulated results calculated by FDTD Lumerical.

In this contribution, the SR was calculated as follows:

(1) $$SR=\frac{SRC}{SIF}$$

where SRC is the current density, defined as the device’s current under illumination, corrected by subtracting the dark current, and normalized by the device’s area. The SIF instead is defined as the power density of the illumination beam on the detector plane, which is measured by means of a calibrated detector. The QE of the device is finally calculated as:

(2) $$QE=\frac{SR}{\lambda }\;\cdot \;\frac{h\;\cdot \;c}{e}$$

where λ is the selected wavelength and $\frac{h\;\cdot \;c}{e}$ is a constant with a value of 1240 $\frac{W\cdot \mathrm{nm}}{A}$. Considering the presented equation, the errors (calculated as ±2σ, therefore a confidence interval of 95%) on the SR and QE measurements are about 6.4% and 7%, respectively, and these values are dominated by the device area error (about 6%) with a wavelength uncertainty (±2σ) of 2.8 nm.

### 4.2. Results and Discussion

**Figure 9:**
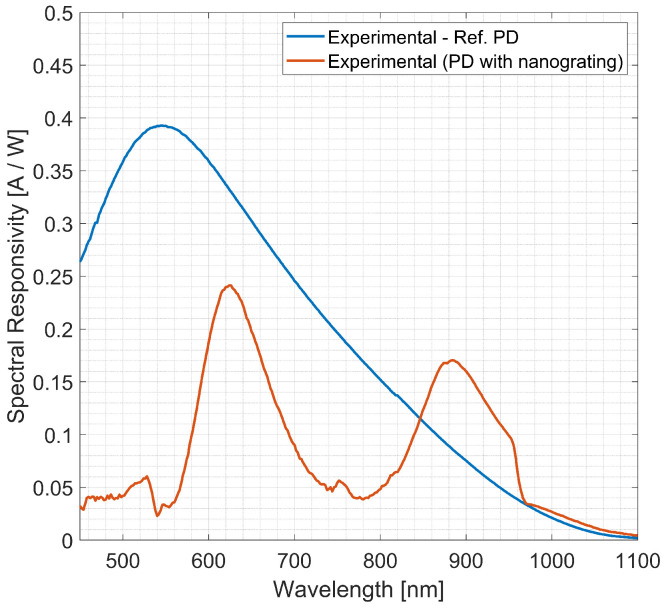
Measured spectral responsivity of a reference PD (blue solid line), measured SR of the integrated PD (orange solid line) as a function of the incident wavelength in the range (450–1100) nm.

**Figure 10:**
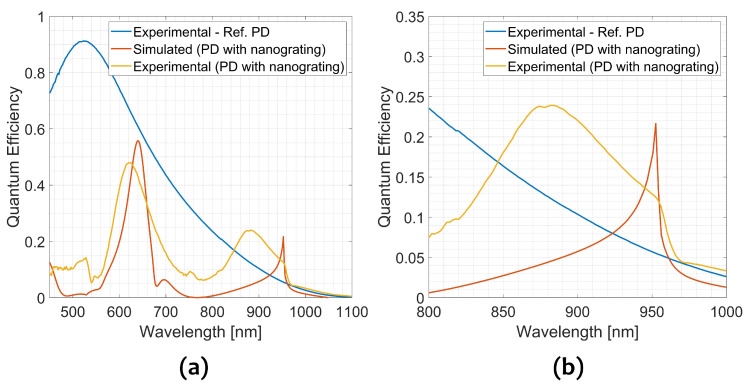
Measured QE of a reference PD (blue solid line), a fraction of absorbed light of the simulated structures (orange solid line) and measured QE of the integrated PD (yellow solid line) as a function of the incident wavelength in the range: (**a**) (450–1100) nm and (**b**) (800–1000) nm. Both simulated and QE of the integrated PD are lower than the QE curve of the reference at almost every wavelength except in the range around the wavelength of our interested 950 nm.

Figure 9 reports the spectral responsivity (SR) of a reference diode (blue solid line) and of the FIB-integrated Si-PD (orange solid line). SR is the current measured [A] at the Si-PD over the incident power [W], and it was measured in the same QE wavelength range 450– 1100 nm. The reference diode’s SR (blue solid line) has a peak at 550 nm with a value of 0.39 $\frac{A}{W}$, reaching a value of SR of 0.045 $\frac{A}{W}$ at 950 nm. The integrated Si-PD’s SR (orange solid line) reproduces all the three main peaks present in the QE: (i) an intense peak of around 0.244 $\frac{A}{W}$ at 620 nm, (ii) a very weak intense peak of 0.055 $\frac{A}{W}$ at 750 nm and (iii) a peak closer to the interesting wavelength of 0.170 $\frac{A}{W}$ at 880 nm. At 950 nm, the SR of reference Si-PD and integrated is of 0.045 $\frac{A}{W}$ and 0.0988 $\frac{A}{W}$, respectively, the latter demonstrating an SR 2.2 times higher than the first. The reported QE and SR curves in Figure 9 and Figure 10 were measured at a constant power value of 1 × 10^−8^ $\frac{W}{{\mathrm{mm}}^{2}}$ at 950 nm. Moreover, variable incident power (from 1 × 10^−8^ $\frac{A}{W}$ to 1 × 10^−6^ $\frac{A}{W}$) was measured by changing the source-to-device distance and the current was measured linearly with the incident power.

Figure 10 reports the measured QE curve of both reference PD and the integrated PD described in this work. The simulated QE of the integrated PD is also reported for comparison (red solid line). In this case, the QE has been calculated from the simulated absorbed power fraction, by considering 100% charge collection efficiency (CCE). In Figure 10, the QE curve of the reference PD is the blue solid line and it has an intense peak of almost 90% at 520 nm, which decreases as the wavelength increases and reaches a value of 5% at 950 nm, the wavelength of our interested. The QE curve of the integrated PD (solid yellow line) reproduces all the three main peaks present in the simulations: (i) an intense peak of around 50% at 620 nm, (ii) a less intense peak of 10% at 750 nm and (iii) a peak closer to the interesting wavelength of 24% at 880 nm.

All the peaks in the measured spectra show slight differences in position or intensity with respect to the simulations. The peak around 620 nm is blueshifted of about 40 nm with respect to its counterpart in the simulated spectrum. In contrast, the less intense peak at 760 nm is redshifted from its counterpart of about 60 nm. Finally, the peak of our interest, which should be at 950 nm, is blueshifted of 70 nm and appears centered at 880 nm, but matching almost perfectly the QE drop at 960 nm. As expected, the QE of the integrated PD is lower than the QE of the reference PD for almost all the measured wavelengths, except for wavelengths around 950 nm, i.e., the target wavelength. At 950 nm the experimental QE shows a value of 12%, 2.2 times more than the reference, but not as high as suggested from simulation (around 22%, thence 4.4 times more).

Probably the difference between expected and measured QE is due to non-idealities found during the characterization of the grating, such as funnel shape slits, Ag roughness, and conformal PMMA profile. The study of these non-idealities on the QE spectra requires further investigation and simulations.

**Figure 11:**
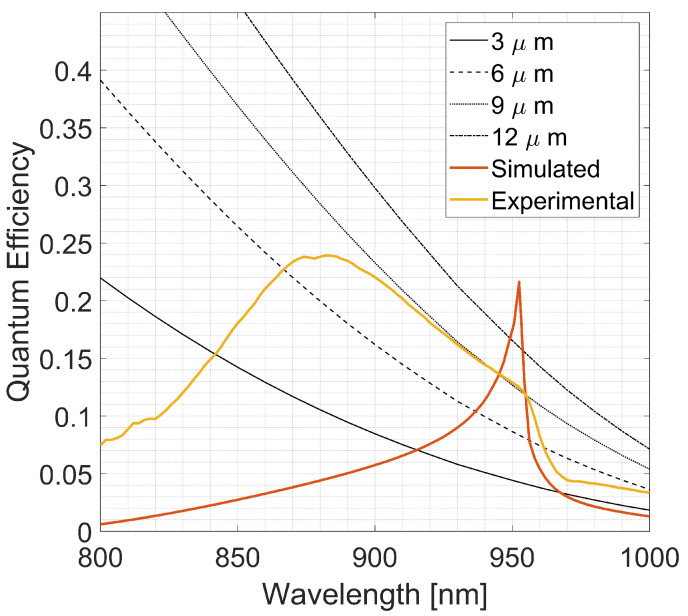
Comparison of the measured QE of the integrated diode and the simulated spectra with the fraction of light absorbed by a slab of silicon covered by an ideal perfect antireflective coating (PARC, null reflection for every wavelength) with increasing thickness. In particular, the QE curve and the simulated spectra are compared to ideal Si slab absorption with the same thickness (3 μm), two- (6 μm), three- (9μm) and fourfold (12 μm) as the PD.

Despite the fabrication-related not-idealities, the experiment QE enhancement can be considered an extremely promising result, demonstrating the strong potential of this structure for NIR absorption enhancement. To confirm this, in Figure 11, the measured QE is compared to the fraction of light absorbed by a slab of silicon covered by an ideal perfect antireflective coating (PARC, null reflection for every wavelength) with increasing thickness.

In particular, the QE curve and the simulated spectra are compared to ideal Si absorption with the same thickness (3 μm), two- (6 μm), three- (9 μm) and fourfold (12 μm) as the PD. It is interesting to note that to obtain the same fraction of absorbed light as one of the integrated diodes at 950 nm, it is necessary to triple the thickness of the Si with an ideal PARC. In addition, if we were able to achieve the same simulated QE, it would mean achieving the same absorption as a 16 μm silicon slab with a device only 3 μm thick.

The obtained performance at 950 nm of integrated Si-PD is not only interesting in terms of NIR light confinement, but also for future technology applications. Indeed, the plasmonic silver array integrated on single-photon detectors like SPADs and SiPMs is necessary for applications which require NIR single-photon sensitivity, low dark count and timing such as LiDAR, time-of-flight (ToF)-imaging, quantum applications (quantum computing, quantum simulations and ghost imaging). In addition, the resonance peak at 950 nm is narrow, and its positions can be changed by modifying the pitch of the plasmonic metal array. This may open to an array of detectors with different resonance peaks position (due to different grating pitch values), useful for spectroscopy applications like high-performance liquid chromatography (HP-LC) and liquid chromatography–mass spectrometry (LC-MS) [36,37].

## 5. Conclusions

Nowadays, light detection and ranging (LiDAR) applications are calling for silicon-based detectors capable of detecting single photons in the near-infrared (NIR). Silicon single photon avalanche diodes (SPADs) could meet the growing demand because of their low fabrication cost, thanks to the well-developed silicon technologies. However, the main drawback of SPADs, the low performance in the NIR region related to the low absorption coefficient of Si in the NIR region, limited their applications.

In this work, we proposed to overcome the limited device’s efficiency by integrating CMOS-like nanostructures, specifically, metal array supporting SPPs, to superficially confine the incoming NIR photons and therefore increase photons’ probability of generating an electron–hole pair. The boost of the Si-PD efficiency in the NIR, specifically at 950 nm, is enabled by taking advantage of the plasmonic confinement of light at a deep-subwavelength scale.

The idea proposed is the integration on the active area of a Si-PD (layers: p++/n-/n++) of a 1-dimensional plasmonic silver array (thickness: 110 nm, pitch: 535 nm, duty cycle: 0.80) over a thin Si_3_N_4_ layer (thickness: 11 nm), passivated by a PMMA layer (thickness: 220 nm). The structure geometry is tuned by FDTD simulations to maximize the absorption of the underlying Si-PD at 950 nm.

From theoretical simulations, we expect an important enhancement (from 5% to 22%) at 950 nm. The Ag plasmonic nanostructures are obtained by patterning an Ag layer by using a Raith Velion FIB-SEM equipped with an Au ion beam.

Finally, the electro-optical properties of the integrated Si-PD with 1D plasmonic silver array are measured. The measured QE curve of the integrated Si-PD presents a peak of 24% at 880 nm. At 950 nm, the experimental QE demonstrates a value of 12%, 2.2 times more than the reference diode without any metal grating (5%) and well beyond the ideal absorption of Silicon with the same thickness, also considering no reflection losses.

Despite the considerable efficiency enhancement at 950 nm, the experimental value is still lower than expected from the simulations. This difference can be due to some non-idealities, such as funnel shape slits, Ag roughness, and conformal PMMA profile. The study of these non-idealities on the QE spectra requires further investigation and simulations.

These results pave the way for further investigation and improvement of the structures, suggesting that even better performance could be achieved in the near future. 

## Data Availability

No new data were created.
